# Pattern recognition receptors in fungal immunity

**DOI:** 10.1016/j.semcdb.2018.03.003

**Published:** 2019-05

**Authors:** Emmanuel C. Patin, Aiysha Thompson, Selinda J. Orr

**Affiliations:** Division of Infection and Immunity, School of Medicine, Cardiff University, Cardiff CF14 4XN, Wales, United Kingdom

**Keywords:** Pathogen recognition receptor, C-type lectin-like receptor, Toll-like receptor, Nod-like receptor, Crosstalk

## Abstract

Over the last decade, invasive fungal infections have emerged as a growing threat to human health worldwide and novel treatment strategies are urgently needed. In this context, investigations into host-pathogen interactions represent an important and promising field of research. Antigen presenting cells such as macrophages and dendritic cells are strategically located at the frontline of defence against potential invaders. Importantly, these cells express germline encoded pattern recognition receptors (PRRs), which sense conserved entities from pathogens and orchestrate innate immune responses. Herein, we review the latest findings regarding the biology and functions of the different classes of PRRs involved in pathogenic fungal recognition. We also discuss recent literature on PRR collaboration/crosstalk and the mechanisms involved in inhibiting/regulating PRR signalling. Finally, we discuss how the accumulated knowledge on PRR biology, especially Dectin-1, has been used for the design of new immunotherapies against fungal infections.

## Highlights

• Growing list of PRRs that sense pathogenic fungal conserved moieties.
• Enhanced immune responses or fungal evasion mechanisms occur through PRR crosstalk.
• Increasing evidence of potent inhibitory mechanisms during PRR signalling.
• PRR targeting strategies to boost immune responses to pathogenic fungi.

## 1 Introduction

Invasive fungal infections have become a major healthcare issue in recent years, with unacceptably high morbidity and mortality levels particularly in immunocompromised patients [1]. Further research in this area is required as there is still no commercial vaccine, the number of antifungal drugs is limited and fungal pathogens are developing drug resistance [2]. A limited number of fungi including *Candida albicans, Aspergillus fumigatus, Cryptococcus neoformans* and *Pneumocystis jirovecii* cause life-threatening conditions in humans [1]. However, invasive fungal infections from other species are currently on the rise including *C. glabrata*, *C. parapsilosis, C. tropicalis* and the multi-drug resistant *C. auris* [3,4]. Therefore, new effective antifungal drugs, immunotherapies and vaccines are urgently needed. An in-depth understanding of host-pathogen interactions is required to help design novel anti-fungal immunotherapies.

Myeloid immune cells such as monocytes, macrophages and dendritic cells (DCs) are the first line of defence during infection. They sense invaders using an array of innate immune receptors termed pattern recognition receptors (PRRs) [5]. PRRs detect conserved structural motifs from microbes and endogenous stress signals called microbe-, pathogen-or damage-associated molecular patterns (MAMPs, PAMPs or DAMPs respectively). The main families of PRRs include Toll-like receptors (TLRs), C-type lectin-like receptors (CLRs), nucleotide-binding oligomerization domain (NOD)-like receptors (NLRs) and retinoic acid-inducible gene-I (RIG-I)-like receptors (RLRs). Following recognition of their respective ligands, these receptors induce innate immune responses for immediate protection or to orchestrate the activation of adaptive immunity [5,6].

Over the last two decades, the biology of PRRs in antifungal immunity has been extensively studied [7,8]. These PRRs sense various fungal cell wall components such as β-glucans, mannans, mannoproteins and chitin as well as fungal-derived RNA and unmethylated DNA (Table 1) [9]. Following ligand binding, PRRs shape immune responses by initiating various signalling cascades which result in fungal internalisation via phagocytosis, cytokine production and/or production of reactive nitrogen and oxygen species (RNS and ROS, respectively) [6,8]. Numerous reports have recently started to unravel the complexity of PRR crosstalk during fungal infections [10,11]. Investigation of potential PRR collaboration/antagonism is essential to develop new therapies to direct the innate immune system towards efficient protection while preventing adverse effects. In this review, we discuss recent findings on PRR-induced anti-fungal immunity with an emphasis on receptor crosstalk/interactions, negative regulation, and the potential for development of novel immunotherapies.

**Table 1:** PRR Recognition of Fungal Pathogens/Ligands.

PRR	Localisation	Cell Types	Motif/Adaptor	Fungal Ligand/Pathogen
CLR				
Dectin-1	Cell surface	Monocytes, MØ, DCs, neutrophils, mast cells, subset of T lymphocytes	hemITAM	ß-glucans
				Zymosan
				*Candida* spp.
				*A. fumigatus*
				*Pneumocystis* spp.
				*Coccidioides* spp.
				*Fonsecaea* spp.
				*T. rubrum*
Dectin-2	Cell surface	Monocytes, MØ, DCs, neutrophils	ITAM-FcRγ	Mannose
				Zymosan
				*Candida* spp.
				*Malassezia* spp.
				*A. fumigatus*
				*P. brasiliensis*
				*Pneumocystis spp.*
				*C. posadasii*
				*F. pedrosoi*
				*H. capsulatum*
				*T. rubrum*
				*C. neoformans*
Mincle	Cell surface	Monocytes, MØ, DCs, neutrophils, mast cells, some subsets of B cells	ITAM-FcRγ	α-mannose
				*C. albicans*
				*A. fumigatus*
				*Pneumocystis* spp.
				*Fonsecaea* spp.
				*Malassezia* spp.
Mcl	Cell surface	Monocytes, MØ, DCs, neutrophils, mast cells	ITAM-FcRγ	*C. albicans*
				*B. dermatitidis*
DC-SIGN	Cell surface	MØ, DCs, activated B cells	Tyrosine-based motif, LSP1	Mannose
				*C. albicans*
				*A. fumigatus*
				*T. rubrum*
MR	Cell surface	MØ, Kupffer cells, endothelial cells	Tyrosine-based motif, FcRγ?	Mannose
				*C. albicans*
				*C. neoformans*
				*P. carinii*
				*C. immitis*
				*P. brasiliensis*
				*H. capsulatum*
				*T. rubrum*

TLR				
TLR2	Cell surface	Monocytes, MØ, DCs, mast cells, neutrophils	MyD88, Mal	Phospholipomannans
				β-glucans
				Zymosan
				*C. albicans*
				*Alternaria*
				*A. fumigatus*
TLR4	Cell surface, Endosome	Monocytes, MØ, DCs, mast cells, neutrophils, B lymphocytes, intestinal epithelium	MyD88, Mal, TRIF, TRAM	*C. albicans*
				*A. fumigatus*
TLR6	Cell surface	Monocytes, MØ, mast cells, B lymphocytes	MyD88, Mal	Zymosan
TLR7	Endosome	Monocytes, MØ, DCs, B lymphocytes	MyD88	*Candida* spp.
TLR9	Endosome	Monocytes, MØ, DCs, B lymphocytes	MyD88	Unmethylated DNA with CpG motif

NLR				
NLRP3	Cytoplasm	Monocytes, DCs, MØ, neutrophils, T and B lymphocytes, epithelial cells	ASC, Caspase-1	*C. albicans*
				*A. fumigatus*
				*C. neoformans*
				*Malassezia* spp
				*P. brasiliensis*
				*S. schenckii*
				*H. capsulatum*
NLRP4	Cytoplasm	DCs, MØ	TBK1	*C. albicans*
NLRP10	Cytoplasm	DCs, MØ, epithelial cells, T lymphocytes	ASC, Caspase-1	*C. albicans*
NOD1	Cytoplasm	Monocytes, DCs, MØ, T and B lymphocytes, intestinal epithelium	RIP2	*A. fumigatus*
NOD2	Cytoplasm	Monocytes, DCs, MØ, T and B lymphocytes	CARD9, RIP2	Chitin
				*C. parapsilosis*
				*A. fumigatus*

RLR				
MDA5	Cytoplasm	Monocytes, DCs, MØ, fibroblasts, epithelial cells, endothelial cells, B lymphocytes	CARDs, MAVS	*C. albicans*

## 2 CLRs

**Fig. 1:**
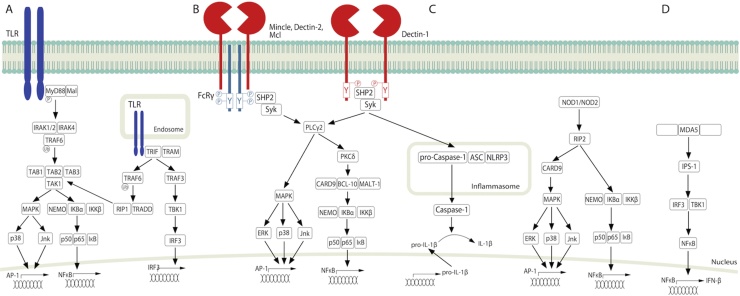
PRR signalling pathways. (A) Following TLR-mediated ligand recognition, MyD88 is recruited to the TLR and a signalling cascade involving IRAK-4, IRAK-1/2, TRAF6, TGF-β-activated kinase 1 (TAK-1), TAK1-binding protein 1 (TAB1), TAB2, TAB3 complex and IκB kinase (IKK)-β is initiated. IKK-β phosphorylates the NFκB inhibitory protein, IκBa, causing its degradation thereby facilitating NFκB nuclear translocation and transcription of proinflammatory cytokines. TLR3, 4, 7, 9, 10 and 13 signal in endosomes via the TRIF-dependent pathway. Ligand-induced TLR activation initiates a TRIF, TRAF3, TBK1 and IRF-3 cascade. Alternatively, TRIF can activate a TRAF6, RIP1, TRADD, TAK-1, NFκB pathway. (B) CLRs signal either by associating with the ITAM-containing FcRγ signalling chain (Dectin-2, Mincle, Mcl) or through a hemITAM in its cytoplasmic tail (Dectin-1). Following ligand recognition, the hemITAM/ITAM are phosphorylated and Syk is recruited. A signalling cascade involving PLCγ, PKCδ and a Card9/Bcl10/Malt1 complex is then initiated. This leads to IKKβ-mediated degradation of IκB to induce nuclear translocation of NFκB (p50, p65). MAPK pathways (ERK, p38 and Jnk) are also activated downstream of PLCγ to induce subsequent AP-1 activation. (C) NLRP3 inflammasome is activated via a CLR-Syk pathway. NLRP3 recruits ASC and pro-Caspase-1 to form an inflammasome complex. This leads to caspase-1 activation and Caspase-1-mediated cleavage of pro-IL-1β into functional IL-1β. Additionally, non-inflammasome forming NLRs such as NOD1 and NOD2 signal via RIP2 to activate the NEMO-IKBα-IKKβ complex to induce p50, p65, IκB and subsequent NFκB activation. In addition, a CARD9-MAPK pathway (ERK, p38 and Jnk) is activated resulting in subsequent AP-1 activation. (D) MDA5 activation by viral RNA, signals through IPS-1 to activate IRF3 and TBK-1, which in turn activates NFκB to induce IFN-β. Whether this occurs in response to fungal pathogens remains to be determined.

Members of the CLR family contain a conserved C-type lectin-like domain (CTLD), which in many cases binds carbohydrates or Ca^2+^ [12]. Some of the best described CLRs during anti-fungal immunity are Dectin-1, the Dectin-2 cluster (Dectin-2, Mincle and Mcl), the mannose receptor (MR) and DC-specific ICAM-3 grabbing non-integrin (DC-SIGN), which are mainly expressed on myeloid cells (Table 1). In addition, collectins such as mannose binding lectin (MBL) and surfactant proteins A and B (SP-A and SP-D) have been reported to play a role in anti-fungal immunity [13]. The roles of these receptors and their signalling pathways have been expertly reviewed elsewhere [6,[13], [14], [15]]. Briefly, Dectin-2, Mincle and Mcl associate with the immunoreceptor tyrosine-based activation motif (ITAM)-containing FcRγ signalling chain while Dectin-1 contains a hemITAM in its cytoplasmic tail. Dectin-2 and Mincle have both been shown to form complexes with Mcl. Following ligand binding the hemITAM/ITAM are phosphorylated, Syk is recruited and canonical NFκB signalling is activated via a Card9/Bcl10/Malt1 complex (Fig. 1). Dectin-1 also activates NFAT and IRF1/5, and a non-canonical NFκB pathway via NFκB inducing kinase (NIK). In addition, DC-SIGN and Dectin-1 both activate RAF1, leading to phosphorylation of TLR or SYK-induced p65 at Ser276. This finely tunes NFκB-induced cytokine responses [6,14,15]. The MR receptor lacks known signalling motifs in its cytoplasmic tail, however it was recently shown to bind the FcRγ signalling chain. MR activation by *Mycobacterium tuberculosis* (*M.tb*) results in recruitment of Grb2 leading to activation of the Rac/Pak/Cdc-42 signalling cascade and recruitment of SHP-1 thereby limiting PI3K activity [16]. Whether this occurs in response to fungal pathogens remains to be determined. While many CLRs are important for anti-fungal immunity we will focus here on Dectin-1 and the Dectin-2 gene cluster.

Dectin-1 binds to ß-glucans from various fungal pathogens (Table 1). Dectin-1 signalling is important for multiple anti-fungal responses including phagocytosis, ROS production, cytokine production, inflammasome activation and Th1 and Th17 responses [6,14,15]. Dectin-1 deficiency has important functional consequences during anti-fungal immunity, fungal allergy and colitis. For example, Dectin-1 KO mice were more susceptible to infections with *C. albicans* [17], *C. glabrata* [18], *A. fumigatus* [19] and *Coccidioides immitis* [20]. In addition, when *C. tropicalis* is present in the gut, Dectin-1 protects against colitis development by limiting fungal invasion [21]. However, in the absence of opportunistic fungi in the gut, inhibition of Dectin-1 protects against colitis due to reduced antimicrobial peptide production resulting in *Lactobacilli murinus* overgrowth and T regulatory cell expansion [22]. Further, during fungal allergy to *A. fumigatus*, Dectin-1 contributes to lung immunopathology by inducing IL-22 [23]. Thus the role of Dectin-1 during fungal-related diseases is complex and context specific. In agreement with various murine studies, patients with Dectin-1 polymorphisms have been shown to have increased susceptibility to mucocutaneous candidiasis [24], aspergillosis [25,26], increased *Candida* colonization [27], increased severity of ulcerative colitis [21] and an altered gut microbiome [28].

Dectin-2 and Mincle bind fungal cell wall mannans (Table 1), and interaction of Mcl with Dectin-2 increased recognition of α-mannans. However distinct hydrophilic and lipophilic ligands from the fungal cell walls of *Malassezia* spp. were identified for Dectin-2 and Mincle, respectively [29]. Dectin-2 is important for anti-fungal immune responses including cytokine production, phagocytosis, ROS production and induction of Th1 and Th17 responses [30] and Mincle is involved in cytokine/chemokine production in response to *Malassezia* spp. and *C. albicans* [31,32]. Dectin-2 deficient mice were more susceptible to infections with *C. albicans* and *C. glabrata* [30,33,34], while Mincle deficient mice displayed increased fungal burdens following infection with *C. albicans* and *P. carinii* [31,35]. Mcl deficient mice developed severe colitis due to defective innate immune responses to *C. tropicalis* in the gut and impaired tissue repair following fungal invasion [36]. In addition, Dectin-2 has been implicated in allergic reactions to house dust mite and *A. fumigatus*. House dust mites and *A. fumigatus* induce production of pro-inflammatory lipid mediators of asthma, such as cysteinyl leukotrienes which in turn induces a Th2 immune response [37,38]. Further, Dectin-2 polymorphisms have been associated with susceptibility to pulmonary cryptococcosis [39] and aspergillosis [26] and polymorphisms in these CLRs have been associated with an altered gut microbiome [28].

## 3 TLRs

TLRs are the best characterised PRR family for pathogen recognition. They are evolutionary conserved transmembrane proteins that sense extracellular and intracellular pathogens in endosomes and lysosomes. The TLRs contain N-terminal leucine-rich repeats (LRRs) and a C-terminal Toll/IL-1R homology (TIR) domain. Most TLRs form homodimers, except TLR2, which forms a heterodimer with TLR1 or TLR6. TLRs signal through the adapter proteins Myeloid differentiation primary response 88 (MyD88), MyD88 adapter-like (Mal), TIR-domain-containing adapter-inducing IFN-β (TRIF) and TRIF-related adaptor molecule (TRAM). The roles of the TLRs and their signalling pathways have been expertly reviewed elsewhere [5,40]. Briefly, TLR signalling usually occurs via two distinct pathways, the MyD88-dependent and the TRIF-dependent pathways (Fig. 1). MyD88 is utilised by all TLRs except for TLR3. Following ligand recognition, MyD88 is recruited to the TLR and a signalling cascade is initiated that culminates in NFκB nuclear translocation and transcription of proinflammatory cytokines (Fig. 1). Endosomal TLRs signal via the TRIF-dependent pathway to induce IRF-3 nuclear translocation and transcription of type I IFN genes (Fig. 1) [5,40].

TLRs play an important role in recognising various fungal pathogens/ligands (Table 1). TLR2 has been shown to be recruited to zymosan-containing phagosomes [41]. In addition, zymosan and various fungal pathogens induce TLR2 or TLR2/TLR6-mediated cytokine responses [[41], [42], [43], [44]]. Further, TLR2 has been shown to induce *C. albicans*-mediated monocyte apoptosis via activation of caspase-8 and caspase-3 [45]. While immunocompetent TLR2, TLR4 and MyD88 deficient mice did not display increased susceptibility to invasive aspergillosis compared to wild type mice [46], another study showed that loss of both TLR2 and TLR4 resulted in severely impaired neutrophil recruitment [47]. SNPs in the TLR2 binding partners, TLR1 and TLR6, have been associated with susceptibility to invasive aspergillosis [26,48]. Interestingly, a synergistic combination of TLR2/6 and TLR9 agonists (Pam2-ODN) as an inhaled treatment protected immunocompromised mice against a broad range of pathogens including *A. fumigatus* [49].

TLR4 recognises O-linked mannosyl chains in the cell wall of *C. albicans* [50]. TLR4 KO mice were more susceptible to systemic infection with *C. albicans* due to reduced chemokine responses and impaired neutrophil recruitment while cytokine responses were normal in TLR4 KO macrophages [51]. In response to *A. fumigatus* conidia but not hyphae, cytokine (TNF, IL-1α, IL-1β) production was reduced in TLR4 KO cells. TLR4-mediated signalling was lost during germination of *A. fumigatus*, which could be a potential means for *A. fumigatus* to evade host innate defences [52]. A link between a TLR4 haplotype and invasive aspergillosis has been suggested by Bochud et al. [53].

TLR7 recognises fungal RNA while TLR9 binds fungal unmethylated CpG DNA (Table 1) [54,55]. Various fungal pathogens/ligands have been shown to induce cytokines (IFN-ß, IFN- α, TNF, IL-12p40) in a TLR7, TLR9 and MyD88-dependent manner [[54], [55], [56]], however TLR9 KO mice were no more susceptible than WT mice to *C. albicans* infection [56] suggesting receptor redundancy. Interestingly, polymorphisms in TLR7, 8 and 9 were recently associated with aspergillosis [26].

## 4 NLRs

NLRs represent another arm of the innate immune system with the ability to detect PAMPs derived from internalised microbial components. The critical function of NLRs/inflammasomes during fungal infections have been expertly reviewed elsewhere [57]. NLRs are structurally composed of a C-terminal ligand sensing series of leucine rich repeats (LRR), a central oligomerisation NACHT domain and an N-terminal protein-protein domain (CARD or pyrin domain). NLRs are categorised into two major family subsets, NOD1/2 and the inflammasome-forming NLRs [58]. Inflammasome multiprotein complexes are generally composed of an NLR such as NLRP3 or NALP3, ASC and Caspase proteins. Inflammasome activation culminates in Caspase-mediated cleavage of pro-IL-1β and pro-IL-18 into functional IL-1β and IL-18 (Fig. 1) [58]. IL-1β and IL-18 have both been reported to be critical for the control of fungal infections [59,60].

Several fungal pathogens have been shown to induce Caspase-1-mediated IL-1β production via NLRP3 (Table 1) [57]. Importantly, it was reported that transition of *C. albicans* from bud to hyphal form was a key event for NLRP3 activation and IL-1β production [61]. In addition, *C. albicans* was shown to induce macrophage cell death via NLRP3-mediated pyroptosis [62]. *A. fumigatus*-induced secretion of IL-1β and IL-18 involved NLRP3, AIM2, ROS production and potassium efflux [63]. Nlrp3, Asc, Caspase-1 KO mice and/or Nlrp3 Aim2 double KO mice were more susceptible to infection with *A. fumigatus*, *C. albicans, C. neoformans, P. brasiliensis* and *S. schenckii* [[63], [64], [65], [66], [67]]. Critically, a polymorphism in NLRP3 was associated with impaired *C. albicans*-induced IL-1β production and increased occurrence of recurrent vulvovaginal candidiasis (RVVC) [68]. Besides NLRP3, the NLRC4 inflammasome was shown to control oral mucosal *C. albicans* infection and to protect against systemic dissemination of *C. albicans* via inflammatory cell recruitment and induction of pro-inflammatory cytokines and antimicrobial peptides [69].

The function of non-inflammasome forming NLRs such as NOD1 and NOD2, during fungal infections has not been investigated as thoroughly as the other NLRs. *A. fumigatus* has been shown to induce expression of NOD1, NOD2 and the downstream signalling molecule RIP2. NOD1 and NOD2 were shown to mediate *A. fumigatus-*induced cytokine release [70,71]. However, NOD1 deficient mice were protected against invasive aspergillosis due to enhanced Dectin-1 expression, ROS and cytokine production [72]. NOD2 polymorphisms were not increased in patients with *Candida* infections and *Candida*-induced cytokine responses were unaffected in patients with NOD2 polymorphisms [73]. NOD2 has since been shown to be involved in recognising chitin and *C. parapsilosis* and inducing downstream cytokine responses [74,75].

## 5 RLRs

The RLR family is composed of three major receptors namely RIG-I, melanoma differentiation factor 5 (MDA5) and laboratory of genetics and physiology 2 (LGP2) which recognise a vast array of RNA viruses and orchestrate innate immune responses mainly through the production of type I IFNs (Fig. 1) [76]. Although extensively studied in the context of antiviral immunity, the involvement of RLRs in anti-fungal immunity has only recently emerged. *C. albicans* hyphae was shown to induce *IFIHI* (MDA5) in macrophages (Table 1) and Mda5 deficient cells produced lower IFN-β levels in response to *C. albicans* compared to WT cells. Furthermore, the study also revealed a strong association with missense variations in the *IFIH1* gene and susceptibility to systemic candidiasis [77]. Further studies are required to fully unravel the complex roles of the CLRs, TLRs, NLRs and RLRs during anti-fungal immunity.

## 6 PRR collaboration/interactions

**Fig. 2:**
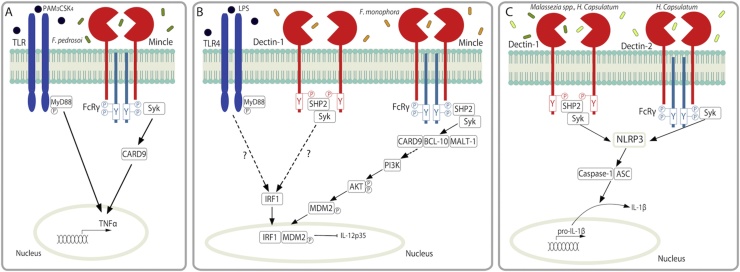
PRR collaboration. (A) *F. pedrosoi* engages Mincle to induce a weak TNF response however artificial engagement of a TLR such as TLR2 by Pam_3_CSK_4_ induces a more robust TNF response. (B) *F. monophora* is recognised by Mincle and leads to CARD9-BCL-10-MALT-1 complex formation through SHP2-Syk activation. This leads to PI3K activation and AKT phosphorylation, rather than NFκB activation. AKT phosphorylates MDM2, which promotes translocation to the nucleus. MDM2 associates with Dectin-1- or LPS-induced IRF1 and the ubiquitin ligase activity of MDM2 is activated. MDM2 targets IRF1 for degradation thereby blocking IL-12p35 activation. (C) *Malassezia spp.* and *H. capsulatum* activate Dectin-1 and Dectin-2 in a Syk-dependent manner to induce NLRP3-mediated IL-1β production through Caspase-1 and ASC signalling to induce Th1/Th17 responses.

Fungal pathogens engage multiple PRRs and interactions between these receptors/pathways can result in an enhanced immune response to clear the pathogen [78], or in some cases can help the pathogen evade the immune response [10]. Some clear examples of PRR interactions in response to *Fonsecaea* spp., the leading cause of the chronic skin condition chromoblastomycosis, have been described. For example, *F. pedrosoi* conidia were shown to induce a weak Mincle-mediated TNF response, however artificial TLR co-engagement induced a more robust TNF response that resulted in improved pathogen clearance (Fig. 2) [79]. In a proof of principle study, the authors demonstrated that topical application of imiquimod, a TLR7 agonist, to the skin lesions of four patients with chromoblastomycosis resulted in a marked improvement of their lesions [80]. In agreement with this, another report showed that *F. pedrosoi* conidia induced a very weak inflammatory response, however the muriform induced a more pronounced pro-inflammatory response. Phagocytosis of the muriform but not the conidia was partially dependent on Dectin-1 and FcγR [81]. While co-engagement of a TLR with *F. pedrosoi* conidia may have therapeutic benefits [78,80], it is possible that co-engagement of a TLR could induce excess inflammation in cases where the muriform is prevalent. Furthermore, Wevers et al. [10] showed that Mincle engagement by *F. monophora* inhibited Th1 cell differentiation. The authors demonstrated that *F. monophora* can induce IL-12p70 production via Dectin-1, however signalling via Mincle counteracts this response and blocks IL12A transcription and subsequent Th1 responses (Fig. 2). Similarly, Shiqueira et al. demonstrated that *F. pedrosoi* conidia and muriform inhibited LPS and IFN-γ-induced IL-12 production [81]. In addition, Wuthrich et al. [82] showed that *F. pedrosoi*-induced Th17 responses were mediated by Dectin-2 and to a lesser extent Dectin-1 while Mincle inhibited these Th17 responses. Unlike the enhanced TNF response observed following co-engagement of a TLR, T cell responses were not augmented [82]. These data indicate that the immune response to *Fonsecaea* spp. involves complex interactions between different PRRs.

Several other examples of PRR crosstalk regulating cytokine production in response to fungal pathogens have been identified. For example, Dectin-1 and Complement receptor 3 (CR3) have been shown to collaborate in response to *H. capsulatum.* Dectin-1 and CR3 co-localised to lipid rafts and induced enhanced activation of a Syk-JNK-AP-1 pathway that resulted in robust TNF and IL-6 production. In addition, Dectin-1 and CR3 cooperated to produce a protective adaptive anti-fungal immune response to *H. capsulatum* in vivo [83]. Dectin-1 has also been shown to form a complex with Galectin-3 resulting in TNF production in response to *C. albicans* [84]. Furthermore, Dectin-1 has been shown to interact with members of the tetraspanin family (CD63 and CD37). CD37 and Dectin-1 co-localised on the surface of antigen presenting cells. Dectin-1-induced IL-6 and IL-6-mediated antibody responses were increased during infection with *C. albicans* in the absence of CD37 resulting in increased pathogen clearance [85,86].

In addition to crosstalk during cytokine responses, PRRs co-operate during ligand recognition and phagocytosis. For example, Dectin-1 was recently shown to control TLR9 trafficking to ß-glucan-, *A. fumigatus*- and *C. albicans*-containing phagosomes [87]. Interestingly, Wu and colleagues linked NOD2 and TLR2 by demonstrating that *A. fumigatus*-induced NOD2 expression was partially mediated by TLR2 [88]. Furthermore, Wagener et al. [75] showed that digested chitin particles are phagocytosed by the mannose receptor, and NOD2 and TLR9 co-localize with internalised chitin leading to the production of IL-10 [75]. In addition, our group recently demonstrated that NOD2 and TLR7 co-localised with internalised *C. parapsilosis* leading to the production of IL-27, although the ligand responsible for this has not yet been identified [74].

Furthermore, CLRs have been linked with inflammasome activation. Dectin-1 induced NLRP3-mediated IL-1β production in response to *Malassezia furfur* (Fig. 2) [89]. In addition, Dectin-1/Syk-mediated inflammasome-dependent IL-1β production in response to *C. albicans* was responsible for inducing Th-17 responses [90]. In agreement with this, another report demonstrated that Caspase-1 and ASC-deficient mice displayed reduced Th1/Th17 responses [91]. Furthermore, *H*. *capsulatum* induced NLRP3 inflammasome-activation and IL-1ß production via Dectin-2. Dectin-1 was not involved in this process in the presence of Dectin-2, however in the absence of Dectin-2, Dectin-1 induced IL-1ß production although to a lesser degree than Dectin-2 [92]. These data clearly demonstrate that fungal pathogens induce co-ordinated PRR-mediated anti-fungal responses.

## 7 Negative regulation of PRR-induced signalling

As with all inflammatory responses, fungal responses require negative feedback mechanisms to control the level of inflammation. Recent reports have identified new feedback mechanisms that are activated during anti-fungal immunity, some of which could be potential therapeutic targets. Various negative regulators of CLRs have been discussed in detail in [11], therefore we will focus here on negative feedback mechanisms that have not been reviewed in detail elsewhere.

**Fig. 3:**
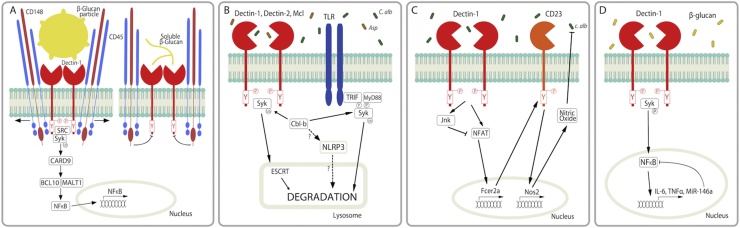
Negative Regulation of PRR-induced signalling. (A) Dectin-1 binds β-glucan particles (such as yeasts) to form the “phagocytic synapse”. The physical interaction between particulate β-glucan and Dectin-1 results in exclusion of CD45 and CD148 tyrosine phosphatases from the synapse. This facilitates Dectin-1 signalling via Src/Syk activation. Soluble β-glucans are unable to exclude the tyrosine phosphatases from the synapse therefore Dectin-1 signalling is blocked by the inhibitory activity of CD45 and CD148. (B) CLR activation results in their ubiquitination and degradation in a Syk-dependent manner. Cbl-b mediates ubiquitination of the activated CLRs through Syk. The ubiquitinated CLRs are then sorted into lysosomes for degradation by an endosomal sorting complex required for transport (ESCRT) system. Additionally, Cbl-b has been shown to target MyD88 and TRIF for degradation following phsophorylation by Syk. Lastly, Cbl-b potentially targets NLRP3 for degradation. (C) *C. albicans* is recognised by Dectin-1, leading to activation of NFAT and Jnk1. NFAT induces CD23 expression and production of nitric oxide. In the absence of Jnk1, NFAT activation, CD23 and nitric oxide levels are increased compared to WT cells. (D) β-glucans induce expression of miR-146a via a Dectin-1-Syk-NFκB pathway. MiR-146a negatively regulates Dectin-1 signalling by supressing NFκB activation.

Dectin-1 binds both soluble and particulate β-glucans, however Dectin-1 signalling is only induced in response to particulate β-glucans. This is due to the formation of a “phagocytic synapse” and exclusion of the tyrosine phosphatases (CD45 and CD148) from this synapse upon binding of particulate β-glucans. Soluble β-glucans are unable to exclude the tyrosine phosphatases therefore Dectin-1 signalling is blocked by the inhibitory activity of these phosphatases (Fig. 3) [93]. As a negative feedback mechanism, phagocytosis of particulate β-glucans following Dectin-1 binding results in reduced Dectin-1 signalling and cytokine responses. Indeed, cytokine production in response to the particulate Dectin-1 ligands, zymosan and curdlan, was significantly increased upon treatment with a phagocytosis inhibitor [94,95]. In agreement with this, poorly immunogenic β-glucan microparticles induced enhanced Dectin-1-mediated cytokine production in the presence of phagocytosis inhibitors [96]. These data indicate that Dectin-1 only responds when it encounters an intact microbe. In addition, delayed phagocytosis due to larger microbe size will likely increase Dectin-1-mediated responses while smaller microbes that are phagocytosed quicker will likely promote weaker Dectin-1-mediated responses.

Three reports independently showed that the E3 ubiquitin ligase Casitas B–lineage lymphoma protein b (Cbl-b) promoted ubiquitination of activated Dectin-1, Dectin-2, Mcl and Syk. The ubiquitinated CLRs were then targeted for lysosomal-mediated degradation, thereby limiting anti-fungal responses (Fig. 3). *C. albicans- and A. fumigatus*-induced cytokine/chemokine and ROS responses were increased in Cbl-b KO cells and mice. Cbl-b KO mice cleared *C. albicans* and *A. fumigatus* infections better than WT mice [[97], [98], [99]]. In addition, Cbl-b targeted MyD88 and Trif for degradation downstream of a TLR-CD11b pathway. In agreement with this, TLR responses were enhanced in the absence of CD11b or Cbl-b [100,101]. It has also been suggested that Cbl-b inhibits NLRP3 inflammasome activation by targeting NLRP3 for ubiquitination although the data supporting this claim is yet unpublished [102]. Additionally, we previously showed that another family member, c-Cbl, is phosphorylated in response to zymosan [103], however whether c-Cbl also targets fungal-related PRRs and associated molecules for degradation is currently unknown. Cbl-b therefore represents a potential therapeutic target during systemic *Candida* and *Aspergillus* infections, however as Cbl-b has many targets, the potential for undesirable side effects such as hyperinflammation must be taken into account.

Interestingly, Zhao and colleagues demonstrated that Jnk1 suppresses anti-fungal immunity through a novel mechanism. A Dectin-1/NFAT pathway induced expression of a CLR, CD23 and production of nitric oxide in Jnk1 deficient macrophages (Fig. 3). Other CLRs and TLRs were largely unchanged in the absence of Jnk1 [104]. Fcer2a, which encodes CD23, is found in a gene cluster with *Cd209a* (DC-SIGN). It is a low affinity receptor for IgE and cystic fibrosis patients with allergic bronchopulmonary aspergillosis have been shown to display increased sensitivity to IL-4 which results in increased CD23 expression on B cells and heightened CD4^+^ Th2 responses to *A. fumigatus* allergens [105]. Zhao et al. showed that in addition to binding IgE, CD23 can also bind both the yeast and hyphal forms of *C. albicans.* Increased CD23 expression and nitric oxide production resulted in increased *Candida* killing and improved survival in Jnk1 deficient mice infected with *C. albicans*. Mouse and human cells treated with Jnk inhibitors displayed increased *Candida* clearance indicating that Jnk1 represents another potential therapeutic target [104].

MicroRNAs (miRNAs) are small noncoding RNAs that regulate gene expression of mRNAs containing complementary sequences. They have been shown to play important roles in regulating inflammatory responses [106]. Some recent reports have identified roles for miRNAs in regulating anti-fungal immune responses and PRR-induced responses. ß-glucan from the *C. albicans* cell wall was recently shown to induce expression of miR-146a, miR-30-5p and miR-210-3p via a Dectin-1-Syk-NFκB pathway. The authors went on to show that miR-146a is a negative feedback regulator of Dectin-1 signalling as miR-146a suppressed Dectin-1-induced NFκB activation and IL-6 and TNF production (Fig. 3) [107]. In contrast, *C. glabrata* has been shown to down-regulate miR-146a expression [108]. Another study showed that heat killed *C. albicans* induced various miRNAs including miR-155, miR-455, miR-125a and miR-146a [109]. In addition, various TLRs involved in antifungal immunity, including TLR2, TLR4, TLR7 and TLR9, have been shown to induce a range of miRNAs such as miR-125a, miR-155, miR-146a, miR-132, miR-147, miR-9, miR-223 and others [[109], [110], [111], [112]]. Various TLR associated molecules have been identified as direct targets of miRNAs such as MYD88, IRAK1/2 and TRAF6 [112]. In addition, miR-125a has been shown to target NOD1 [113] while miR-223 and miR-9 suppress NLRP3 expression/activation [114,115]. Clearly, miRNAs are involved in regulating the PRR-induced pathways during anti-fungal immunity, however, further study is required in this area.

## 8 Harnessing PRRs for the development of novel immunotherapies against fungal infections: the case of Dectin-2

One of the ultimate goals behind investigations into host-pathogen interactions is to identify new immunotherapy strategies. This may involve stimulation or interference with PRR signalling or specifically targeting fungal antigens to DCs to improve immune-mediated protection. This is particularly relevant for vaccine research where extensive efforts have been undertaken to design better vaccine delivery tools and novel adjuvants with increased immune reactivity and lower toxicity. Numerous investigations have assessed the potential of fungal cell wall components to induce protective immunity to various diseases including infectious and autoimmune diseases as well as cancer. In particular, Dectin-1 agonists such as β-glucans have been extensively studied for their potent immunostimulatory properties. In addition, antibodies against Dectin-1 have been used to target fungal antigens to DCs and T cells have been modified to express a Dectin-1-chimeric antigen receptor. These could represent interesting strategies for future immunotherapies against fungal infections. A full description of all the studies in this area is beyond the scope of this review, therefore we will focus on select fungal-related studies.

Various in vitro studies have demonstrated the potential of β-glucan preparations to enhance immune responses to fungal pathogens. For example, curdlan has been shown to boost *A. fumigatus*-induced pro-inflammatory cytokine production and β-glucan was shown to amplify microbial toxicity of human neutrophils to both *C. albicans* and *C. glabrata* [116,117]. Similarly, several in vivo studies demonstrated the therapeutic potential of β-glucan administration. In combination with normal antifungal treatments, intravenous administration of β-glucan to patients infected with *P. brasiliensis* resulted in reduced serum antibody levels and increased *P. brasiliensis*-associated CD4^+^ T cell responses [118]. In addition, the β-glucan Laminarin conjugated to the diphtheria toxoid CRM197 was shown to induce protection in mice systemically infected with *C. albicans* via the induction of high levels of β-glucan antibodies [119]. in vivo imaging studies also revealed that treatment with the same Laminarin-CRM vaccine-conjugate, in combination with MF59 adjuvant, could protect mice against vaginal *C. albicans* infection [120]. Additionally, another study showed that Laminarin-conjugates could ameliorate antibody-mediated immunity to *C. albicans* in mice [121]. Oral administration of β-glucan has also been shown to increase protection against intestinal inflammation and *C. albicans* colonisation in mice [122]. Another interesting study showed that administration of whole glucan particles (WGP) could significantly increase survival of mice challenged with *A. fumigatus*. This was associated with reduced fungal burden in both brain and kidney and elevated cytokine expression in the WGP treated mice [123]. Follow-up studies from the same group showed that WGP-vaccinated mice challenged intravenously with *Coccidioides posadasii* had reduced mortality rates and lower fungal burden in the lung, liver and spleen [124]. Taken together, these findings highlight Dectin-1 agonists as promising candidates for the development of immunotherapies to treat fungal infections in humans. However, it remains to be elucidated whether treatments using β-glucan in combination with selected activators or inhibitors of other PRR pathways could represent an interesting strategy to further enhance immune protection.

Some groups have evaluated the potential of Dectin-1 antibodies as a delivery system to specifically target antigens such as ovalbumin and haemagglutinin to DCs and subsequently increase protective immune responses to infectious agents. Anti-Dectin-1-antigen conjugates have been shown to trigger potent CD4^+^ and CD8^+^ T cell responses [[125], [126], [127]]. This therefore represents an interesting strategy to improve vaccine efficacy to microbial pathogens. However, it remains to be determined whether this approach will have any impact against fungal pathogens.

Finally, CAR T cell technology was recently exploited to generate cytotoxic T cells specific for fungal pathogens. A novel Dectin-1-chimeric antigen receptor (D-CAR) was bioengineered using the extracellular domain of Dectin-1 to redirect T-cell specificity towards fungal β-glucan moieties [128]. It was shown that D-CAR^+^ T cells could inhibit *A. fumigatus* hyphae formation in vitro through recognition of β-glucan. In addition, immunosuppressed mice treated with D-CAR^+^ T cells exhibited reduced pulmonary *A. fumigatus* burden when compared with mice administered with control CD19-specific CAR^+^ T cells. Thus, these findings represent a promising step for the development of novel efficient immunotherapies using host-pathogen interactions to control fungal infections in immunocompromised individuals.

## 9 Conclusions

Collaborative efforts from the scientific community have provided an important body of literature on PRR biology that will be extremely valuable for the development of immunotherapies to treat fungal infections. In recent years, it has become evident that PRR crosstalk and inhibitory feedback mechanisms help finely tune anti-fungal immune responses. While much progress has been made to identify PRR crosstalk and inhibitory mechanisms, further work is required to fully unravel the complexities of these PRR interactions. A better understanding of PRR biology/interactions will be invaluable to help develop immunotherapies for fungal infections and other inflammatory diseases.

## Funding

SJO is funded by a Sir Henry Dale Fellowship jointly funded by the Wellcome Trust and the Royal Society (Grant Number 099953/Z/12/Z).
